# Patellofemoral Joint Loading Progression Across 35 Weightbearing Rehabilitation Exercises and Activities of Daily Living

**DOI:** 10.1177/03635465231175160

**Published:** 2023-06-05

**Authors:** Ke Song, Rodrigo Scattone Silva, Todd J. Hullfish, Karin Grävare Silbernagel, Josh R. Baxter

**Affiliations:** † Department of Orthopaedic Surgery, University of Pennsylvania School of Medicine, Philadelphia, Pennsylvania, USA; ‡ Department of Physical Therapy, University of Delaware, Newark, Delaware, USA; § Postgraduate Program in Rehabilitation Sciences, Postgraduate Program in Physical Therapy, Federal University of Rio Grande do Norte, Santa Cruz, Brazil; Investigation performed at the University of Pennsylvania School of Medicine, Philadelphia, Pennsylvania, USA

**Keywords:** biomechanics, motion analysis, knee, patellofemoral pain, anterior knee pain, physical therapy

## Abstract

**Background:**

Exercises that provide progressive therapeutic loading are a central component of patellofemoral pain rehabilitation, but quantitative evidence on patellofemoral joint loading is scarce for a majority of common weightbearing rehabilitation exercises.

**Purpose:**

To define a loading index to quantify, compare, rank, and categorize overall loading levels in the patellofemoral joint across 35 types of weightbearing rehabilitation exercises and activities of daily living.

**Study Design:**

Descriptive laboratory study.

**Methods:**

Model-estimated knee flexion angles and extension moments based on motion capture and ground-reaction force data were used to quantify patellofemoral joint loading in 20 healthy participants who performed each exercise. A loading index was computed via a weighted sum of loading peak and cumulative loading impulse for each exercise. The 35 rehabilitation exercises and daily living activities were then ranked and categorized into low, moderate, and high “loading tiers” according to the loading index.

**Results:**

Overall patellofemoral loading levels varied substantially across the exercises and activities, with loading peak ranging from 0.6 times body weight during walking to 8.2 times body weight during single-leg decline squat. Most rehabilitation exercises generated a moderate level of patellofemoral joint loading. Few weightbearing exercises provided low-level loading that resembled walking or high-level loading with both high magnitude and duration. Exercises with high knee flexion tended to generate higher patellofemoral joint loading compared with high-intensity exercises.

**Conclusion:**

This study quantified patellofemoral joint loading across a large collection of weightbearing exercises in the same cohort.

**Clinical Relevance:**

The visualized loading index ranks and modifiable worksheet may assist clinicians in planning patient-specific exercise programs for patellofemoral pain rehabilitation.

Patellofemoral pain is one of the most common musculoskeletal conditions among physically active adolescents and young adults,^10,35,39^ with an annual prevalence of 23% in the general population.^33^ Patellofemoral pain is not a self-limiting condition, and it reduces the physical activity levels of affected individuals.^21,30^ Patellofemoral pain may be associated with the development of patellofemoral osteoarthritis,^36^ which highlights the importance of appropriate treatment strategies for young, active patients to preserve long-term joint health.

Exercise is the cornerstone of rehabilitation for patellofemoral pain. State-of-the-art rehabilitation strategies can reduce patellofemoral pain and improve patient function in the short term.^8,40^ However, research has shown that long-term prognosis of patellofemoral pain is still poor, as 30% to 50% of patients report unfavorable recovery at 5- to 20-year follow-up.^9,21,25^ A recent systematic review suggested that current rehabilitation exercise programs may be too simplistic to address the key pathomechanical aspects of patellofemoral pain.^13^ Most rehabilitation programs in clinical trials have focused on open kinetic chain exercises with isolated knee movements in nonweightbearing positions.^13^ In contrast, weightbearing exercises that involve multiplanar movements are underused in rehabilitation,^13^ and their contributions to patellofemoral joint mechanics and biological health need to be better understood.

The current pathomechanical model of patellofemoral pain suggests that abnormal patellofemoral joint loading during exercises and activities of daily living is a direct contributor to symptoms.^29^ Therefore, understanding patellofemoral joint loading during common exercises and activities is essential for implementing rehabilitation plans that can provide therapeutic loading progression to the joint. Previous biomechanical studies have evaluated patellofemoral joint peak force and stress during daily living activities such as walking,^4,23^ running,^22^ stairs,^3,6^ and a few common exercises including jumping,^7^ squats,^17,28,38^ and lunges.^15,16,18^ Yet patellofemoral joint loading profiles remain unknown for a majority of exercises commonly used in patellofemoral or anterior knee pain rehabilitation.^1,5,11,13,27,35,40^ Additionally, no studies have reported how patellofemoral joint loading during the large collection of rehabilitation exercises compares with routine walking and running in the same individuals. Due to the lack of comparable evidence, clinicians often need to rely on generalized experience and intuition when developing rehabilitation exercise protocols. More quantitative data are needed to inform patellofemoral joint rehabilitation strategies that suit patient-specific recovery needs.

To establish quantitative guidelines that prescribe progressive therapeutic loading during rehabilitation, one useful approach is to rank and categorize exercises based on how they load the patellofemoral joint. Willy and Meira^41^ suggested that it may be important to characterize patellofemoral joint loading profiles beyond peak magnitude alone. Specifically, attention should be paid to the cumulative patellofemoral joint loading over time when developing rehabilitation plans for patellofemoral pain. In a recent study,^2^ we developed a loading index system to characterize loading progression in the Achilles tendon across 25 types of common rehabilitation exercises, which can be customized by clinicians to adjust exercise protocols for patient-specific rehabilitation. This loading index system is based on a comprehensive data set that factored not only loading peak but also loading rate and the cumulative loading impulse. However, such a complete loading data set is missing for many commonly prescribed weightbearing patellofemoral joint exercises relevant to physically active individuals with patellofemoral pain. Establishing this data set is necessary to develop a similar multifactorial and adjustable loading index system for the patellofemoral joint.

The goal of our study was to establish a loading index to quantify, compare, rank, and categorize overall loading levels in the patellofemoral joint across 35 types of commonly prescribed weightbearing rehabilitation exercises and activities of daily living. These loading guidelines may provide clinicians with new evidence to progressively load the patellofemoral joint, which could potentially improve functional recovery in individuals with patellofemoral pain.

## Methods

### Participants and Study Preparation

We recruited 20 healthy adult participants (10 male, 10 female; age, 25.9 ± 5.7 years; body mass index, 24.1 ± 2.6) between January and June 2022 from the university campus and local community. To be included, participants had to be between 18 and 40 years of age and have no self-reported history of injury in the lower limbs or spine in the last 6 months. An experienced physical therapist (R.S.S.) confirmed that each participant had no current anterior knee pain or history of patellofemoral joint injury. We excluded those with previous injuries or current pain in order to eliminate the confounding effects of compensatory movements on patellofemoral biomechanics. Participants provided written informed consent before participating, and the study was approved by the University of Pennsylvania’s institutional review board.

**Figure 1.**
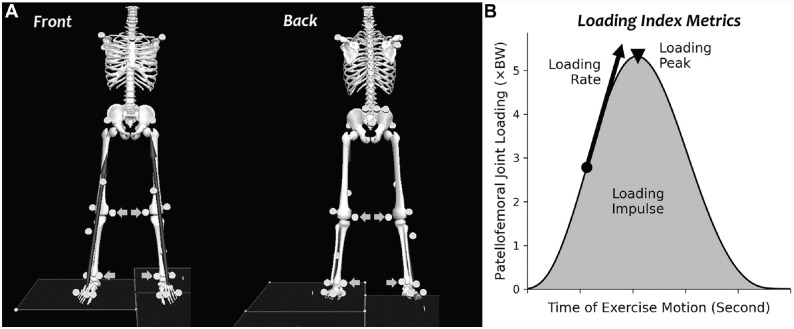
(A) Marker set to track the positions of the pelvis, lower extremities, and torso. We removed calibration-only markers (arrows) after an anatomic pose trial. (B) The 3 loading index metrics were defined in our recent study^2^: loading peak, loading impulse, and loading rate. BW, body weight.

Participants wore standard exercise clothing (running shorts and tank tops) and running shoes (Air Pegasus; Nike). A single examiner (K.S.) placed 31 retroreflective markers over anatomic landmarks of the pelvis, both thighs, shanks, feet (on the shoes), and upper torso to track body segment positions (Figure 1A).^32^ We first recorded a static trial with the participant standing in an anatomic pose, and then we removed 4 calibration-only markers on the medial knees and ankles so these markers would not interfere with each participant’s natural movement during exercises.

### Data Collection and Processing

Each participant performed 35 weightbearing exercises that are either routine activities of daily living or common in physical therapy rehabilitation for patellofemoral or anterior knee pain.^1,5,13,27^ We divided the exercises into 4 modules to minimize the potential effects of physical exhaustion. Each exercise module included 7 to 10 trials with various levels of intensity (detailed descriptions are provided in Appendix Table A1, available in the online version of this article), and we provided 3- to 5-minute rest periods between the modules. We visually confirmed during pilot testing that participants would not be physically exhausted by this exercise order. All participants performed the exercises in the same order. Participants practiced each exercise after being provided visual demonstrations. Participants received verbal guidance throughout each trial by the same physical therapist, who has >10 years of clinical experience (R.S.S.). We collected ≥3 successful repetitions for each exercise, including 3 pairs of back-and-forth motions during exercises such as alternating lateral or split jumps. We simultaneously recorded marker trajectories using a 12-camera motion capture system at 100 Hz (Raptor Series; Motion Analysis Corp.) and ground-reaction forces using 3 force platforms embedded under the floor at 1000 Hz (BP600900; AMTI).

We manually confirmed that motion capture marker trajectories were correctly labeled to ensure continuous tracking of the pelvis and lower body segments. We filled small gaps of marker trajectories (<0.3 seconds) using cubic splines and imported labeled trajectories into a biomechanical model (Visual3D; C-Motion). We used a constrained link-segment model to solve for best estimates of segment positions.^32^ This model constrained the knee joint to flexion-extension motion only, while allowing the hip and ankle joints to have all 3 rotational degrees of freedom. Our recent study^32^ demonstrated that this model can track segment positions with high fidelity with the simple marker set we used. We verified that the ground-reaction forces were assigned to the correct model foot in all recorded trials, including through accessories such as stepping boxes. We extracted individual exercise repetitions within each trial using the ground-reaction forces or manually defined start and stop events (definition details provided in Appendix Table A1, available online) for the subsequent biomechanical analysis.

### Biomechanical Analysis

To quantify patellofemoral loading, we first calculated right knee joint flexion angles and extension moments from the link-segment model using inverse kinematic and inverse dynamic algorithms.^31^ We chose the right leg as the side of interest for each participant. We applied a fourth-order, bidirectional, Butterworth low-pass filter with a 6-Hz cutoff frequency to the knee angle and moment estimates to reduce nonphysical components due to motion capture system artifacts and force platform signal noise.^31^

We computed the patellofemoral joint forces during each exercise repetition using knee flexion angles, extension moments, and a mathematical model of knee moment arm lengths and force relationships.^3,4,37^ This model-based computation involves several steps. To begin, computing the patellofemoral joint force requires an estimation of force in the quadriceps tendon. Because physical moment arms for the quadriceps tendon are ambiguous to define, we estimated the knee flexion-dependent quadriceps tendon force using a previously established “effective” quadriceps tendon moment arm (Equations 1 and 2).^3,4^ Then we estimated the patellofemoral joint force according to its relationship to the quadriceps force (termed the “k-constant”) reported in the same literature (Equations 3 and 4).^3,4,37,42^ In the following equations, *quad* refers to the quadriceps tendon; *PFJ*, patellofemoral joint; *MA*, moment arm; *F*, force; *θ*, knee flexion angle (in degrees); and *t*, time.

Equation 1 $$MA_{\mathrm{Quad}}\left(\theta_{t}\right)=8.0e^{-5}{\theta_{t}}^{3}-0.013{\theta_{t}}^{2}+0.28\theta_{t}+46\left(\mathrm{unit}:\mathrm{mm}\right).$$

Equation 2 $$F_{\mathrm{Quad}}\left(\theta_{t}\right)=M_{\mathrm{knee}}\left(\theta_{t}\right)/MA_{\mathrm{Quad}}\left(\theta_{t}\right)\left(\mathrm{unit}:\mathrm{Newton}\right).$$

Equation 3 $$k\left(\theta_{t}\right)=\frac{-3.84e^{-5}{\theta_{t}}^{2}+1.47e^{-3}\theta_{t}+0.462}{-6.98e^{-7}{\theta_{t}}^{3}+1.55e^{-4}{\theta_{t}}^{2}-0.0162\theta_{t}+1}\left(\mathrm{unitless}\right).$$

Equation 4 $$F_{\mathrm{PFJ}}\left(\theta_{t}\right)=k\left(\theta_{t}\right)*F_{\mathrm{Quad}}\left(\theta_{t}\right)\left(\mathrm{unit}:\mathrm{Newton}\right).$$

The patellofemoral joint and quadriceps forces described above are part of our larger mathematical scheme to quantify loading in the anterior knee structural complex. This scheme of equations also includes 2 knee tendons of clinical importance—the patellar and quadriceps tendons. Thus, we additionally present the equations derived from up-to-date data^12,37^ to estimate patellar tendon moment arm and force (Equations 5 and 6), as a unified point of reference for future biomechanics research on the anterior knee structural complex.

Equation 5 $$MA_{\mathrm{PT}}\left(\theta_{t}\right)=4.66e^{-5}{\theta_{t}}^{3}-9.43e^{-3}{\theta_{t}}^{2}+0.397\theta_{t}+46.7\left(\mathrm{unit}:\mathrm{mm}\right).$$

Equation 6 $$F_{\mathrm{PT}}\left(\theta_{t}\right)=M_{\mathrm{knee}}\left(\theta_{t}\right)/MA_{\mathrm{PT}}\left(\theta_{t}\right)\left(\mathrm{unit}:\mathrm{Newton}\right).$$

We normalized the estimated patellofemoral joint forces (Equation 4) by participant body weight (BW). We zeroed all negative force components that resulted from knee moment because patellofemoral joint loading is physically one-way (ie, articular cartilage contact force can only be compression, not tension). Next, we computed 3 loading metrics on the estimated forces following our earlier methods^2^: loading peak as the maximum force in each repetition, loading impulse as the area under the force-time curve over each repetition, and loading rate as the maximum instantaneous change of force over time (Figure 1B). We averaged each loading metric across exercise repetitions within each trial and included pairs of alternating motions where the right leg was abducted/adducted or in a leading/trailing position. We then calculated the group means and standard deviations of each loading metric (peak, impulse, rate) during each of the 35 exercises. We also computed the peak, impulse, and rate of knee flexion angles and extension moments during each exercise to compare with literature and interpret our loading results. We provide the group means of these data in the Appendix (available online).

We used the group means of the 3 loading metrics to compute a weighted loading index as described in our recent study.^2^ Briefly, we normalized the loading peak, impulse, and rate by the maximum of each among the 35 exercises. We then computed a weighted sum with a 50% weight on loading peak and 50% on loading impulse, resulting in 1 loading index for each exercise. We chose these weights based on our own clinical intuition of current research concepts.^29,41^ Specifically, we assigned equal weights to loading peak and impulse considering the need for emphasis on both cumulative and peak loading,^41^ whereas no weight was placed on loading rate because there is no current research consensus on its influence on patellofemoral pathomechanics.^29^ Each loading index theoretically ranges from 0 to 1, with 0 representing no load and 1 representing an exercise that would have loading peak and impulse both reaching maximum. We ranked the 35 loading indices in a progressive ascending order and categorized them into 3 equally divided tiers: tier 1 (low, <0.333), tier 2 (moderate, 0.333-0.667), and tier 3 (high, >0.667). We also embedded the group-average loading metrics into a modifiable worksheet (see Appendix, available online), so anyone can rerank and recategorize the loading indices based on user-selected weights on the loading metrics, including the loading rate if desired.

## Results

**Figure 2.**
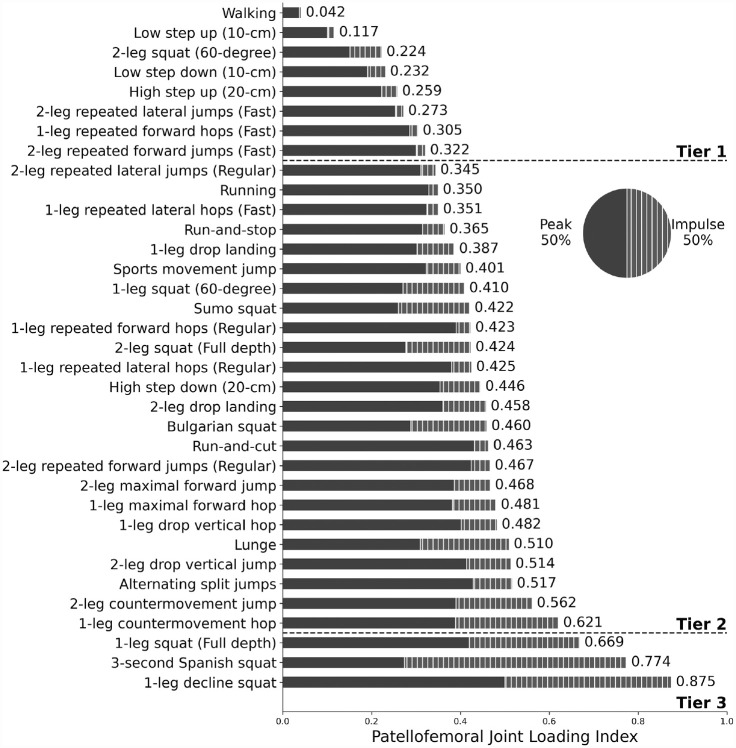
Patellofemoral joint loading indices ranked in ascending order across 35 rehabilitation exercises and daily living activities, categorized as low (tier 1), moderate (tier 2), and high (tier 3). Each loading index was summed with a 50% weight on loading peak (solid) and 50% on loading impulse (striped).

Exercise loading indices ranged from the lowest of 0.042 during walking to the highest of 0.875 during the single-leg decline squat (Figure 2). Tier 1 exercises (loading index < 0.333) included walking, 60° double-leg squat, most stepping exercises, and some jumping and hopping exercises. Peak patellofemoral loading in tier 1 exercises ranged from 0.6 to 4.9 ×BW (Table 1). Of the 35 exercises, 24 exercises (69%) fell into tier 2 (0.333 < loading index < 0.667). Tier 2 exercises included running, all jumping and hopping exercises that were not in tier 1, lunges, and most squats. Peak loading in tier 2 exercises ranged from 4.3 to 7.1 ×BW (Table 1). Tier 3 included 3 challenging variations of squatting exercises: the full single-leg squat (0.669), 3-second Spanish squat (0.774), and single-leg decline squat (0.875). Peak loading ranged from 4.5 to 8.2 ×BW among these tier 3 exercises (Table 1).

**Table 1:** Group-Level (N = 20) Values for Patellofemoral Joint Loading Metrics Across the 35 Exercises and Activities, Listed in Ascending Order of the Loading Index^*a*^

Exercises in Each Loading Index Tier	Peak, ×BW	Impulse, ×BW·s	Rate, ×BW/s
Tier 1			
Walking	0.6 ± 0.3	0.1 ± 0.1	9.4 ± 4.1
Low step-up (10 cm)	1.7 ± 0.4	0.4 ± 0.1	13.9 ± 3.5
Double-leg squat (60°)	2.5 ± 0.9	1.9 ± 0.7	6.3 ± 2.9
Low step-down (10 cm)	3.1 ± 0.8	1.0 ± 0.5	14.4 ± 4.2
High step-up (20 cm)	3.6 ± 0.6	0.9 ± 0.2	30.2 ± 5.5
Double-leg repetitive lateral jumps (fast)	4.2 ± 1.6	0.5 ± 0.2	56.0 ± 18.8
Single-leg repetitive forward hops (fast)	4.7 ± 1.9	0.5 ± 0.3	84.2 ± 25.4
Double-leg repetitive forward jumps (fast)	4.9 ± 2.2	0.5 ± 0.3	82.3 ± 31.7
Tier 2			
Double-leg repetitive lateral jumps (regular)	5.1 ± 1.5	0.9 ± 0.4	57.3 ± 12.7
Running	5.4 ± 1.2	0.5 ± 0.2	87.6 ± 17.2
Single-leg repetitive lateral hops (fast)	5.3 ± 1.8	0.7 ± 0.3	72.5 ± 20.5
Run-and-stop	5.2 ± 1.4	1.3 ± 0.6	86.0 ± 22.3
Single-leg drop landing	4.9 ± 1.3	2.2 ± 1.1	62.0 ± 14.2
Sports movement jump	5.3 ± 1.3	2.0 ± 0.9	80.8 ± 21.8
Single-leg squat (60°)	4.4 ± 1.2	3.6 ± 1.1	10.8 ± 4.3
Sumo squat	4.3 ± 0.5	4.2 ± 1.0	15.8 ± 3.1
Single-leg repetitive forward hops (regular)	6.4 ± 1.8	0.8 ± 0.3	91.4 ± 19.0
Double-leg squat (full depth)	4.5 ± 0.8	3.8 ± 0.8	14.8 ± 4.1
Single-leg repetitive lateral hops (regular)	6.2 ± 1.7	1.2 ± 0.4	69.1 ± 14.6
High step-down (20 cm)	5.8 ± 0.9	2.4 ± 1.2	21.1 ± 5.5
Double-leg drop landing	5.9 ± 1.2	2.5 ± 1.0	73.1 ± 16.3
Bulgarian squat	4.7 ± 0.7	4.4 ± 1.0	15.4 ± 4.1
Run-and-cut	7.1 ± 2.1	0.8 ± 0.4	115.7 ± 27.8
Double-leg repetitive forward jumps (regular)	6.9 ± 1.3	1.1 ± 0.4	96.1 ± 18.2
Double-leg maximal forward jump	6.3 ± 0.8	2.1 ± 0.7	95.3 ± 14.3
Single-leg maximal forward hop	6.3 ± 1.2	2.6 ± 1.4	93.1 ± 18.0
Single-leg drop vertical hop	6.6 ± 1.8	2.1 ± 0.7	73.8 ± 19.9
Lunge	5.1 ± 0.8	5.2 ± 1.3	20.4 ± 6.4
Double-leg drop vertical jump	6.8 ± 1.4	2.6 ± 0.7	75.4 ± 17.7
Alternating split jumps	7.0 ± 1.6	2.3 ± 0.6	60.7 ± 16.8
Double-leg countermovement jump	6.4 ± 1.1	4.5 ± 1.6	72.5 ± 16.2
Single-leg countermovement hop	6.4 ± 1.6	6.0 ± 2.0	69.6 ± 24.0
Tier 3			
Single-leg squat (full depth)	6.9 ± 1.1	6.4 ± 1.4	17.8 ± 4.7
3-second Spanish squat	4.5 ± 1.6	13.0 ± 4.6	2.5 ± 2.0
Single-leg decline squat	8.2 ± 1.0	9.7 ± 3.0	19.3 ± 4.7

a Values are expressed as mean ± SD. BW, body weight.

Loading peaks, impulses, and rates all varied substantially among the 35 exercises, including some that ranked similarly according to the loading index (Figure 2; Table 1). For example, loading index was similar between run-and-cut (0.463) and the Bulgarian squat (0.460). However, the loading index of run-and-cut was mostly explained by loading peak, whereas the loading index of the Bulgarian squat had a substantial contribution of loading impulse (Figure 2). Notably, none of the 35 exercises reached a loading index >0.9 despite the theoretical possibility. This is likely because the exercise that presented the highest loading impulse by far (3-second Spanish squat, 13.0 ×BW·s) had a lower loading peak (4.5 ×BW) than the other tier 3 exercises (Table 1), and no exercise had a loading peak and loading impulse that reached the highest level at the same time.

All 3 exercises categorized into tier 3 are challenging variations of squatting exercises, which involve a relatively high degree of knee flexion and movement duration. Conversely, many fast exercises such as running (0.350; tier 2) and single-leg repeated fast forward hops (0.305; tier 1) provided a relatively low to moderate level of overall patellofemoral loading.

## Discussion

We defined a loading index to quantify, compare, rank, and categorize overall loading levels in the patellofemoral joint across 35 types of commonly prescribed weightbearing rehabilitation exercises and activities of daily living. To the best of our knowledge, the biomechanics of many of these exercises had not been rigorously analyzed in the past. The visualized loading index ranks and tiers (Figure 2) provide a straightforward reference for clinicians to compare rehabilitation exercises that biomechanically resemble each other. These loading guidelines may allow clinicians to refine patellofemoral pain rehabilitation programs that use progressive therapeutic loading for better treatment outcomes while also suiting each patient’s needs and functional capacities. We also provide our group-level data in a modifiable worksheet (see Appendix, available online) that automatically reranks and recategorizes exercises according to the relative importance assigned to the loading peak, impulse, and rate by a clinician. Finally, our larger mathematical scheme to quantify anterior knee structural loading sets a benchmark for future research to identify the mechanical biomarkers of knee diseases in active individuals, including the mechanistic relationships between patellofemoral pain and other knee pathologies.

According to the loading index, most rehabilitation exercises generate a moderate level of patellofemoral joint loading (tier 2). Few weightbearing exercises provide low-level loading that resembles routine walking (tier 1) or high-level loading that features both high magnitude and long duration (tier 3). The abundance of exercises with moderate loading suggests that clinicians may simplify rehabilitation protocols for patellofemoral pain into a few common exercises. Specifically, clinicians may choose exercises that best suit patient needs and functional capacities during late rehabilitation stages when patellofemoral joint loading has progressed to a moderate level, as patients prepare to resume sports and everyday exercises such as running. Conversely, the scarcity of weightbearing exercises that provide low-level loading suggests that even for patients who are symptom-free during walking, clinicians should be cautious against progressing to further weightbearing too early to avoid overloading the joint.^27^ Powers et al^28^ reported that seated knee extension generates peak patellofemoral stress similar to a 60° double-leg squat—a tier 1 exercise (Table 1)—and can be gradually reduced toward zero by placing the knee in a more flexed position.^28,34^ During the initial stage of rehabilitation, such open kinetic chain exercises may be more tolerable and feasible for providing a subwalking level of loading.^28^ Among the 35 exercises we analyzed, only the low step-up provides patellofemoral joint loading with a loading index <0.2, and only 3 other exercises (60° double-leg squat, low step-down, high step-up) provide further progressive loading below any sports-related activities (eg, fast lateral jumps). It may thus be important to prescribe these exercises in early to midterm rehabilitation to progress the joint’s weightbearing capacity beyond walking, thereby gradually improving a patient’s envelope of function.^14,41^ On the other end, the Spanish squat and single-leg decline squat are the only exercises in tier 3 that ranked higher than a full single-leg squat. Considering that these 2 squatting variations are not common functional activities or everyday exercises, they may be useful exclusively as last-stage rehabilitation exercises for patients who are routinely submitted to high patellofemoral joint loads in sports or work-related activities.

**Figure 3.**
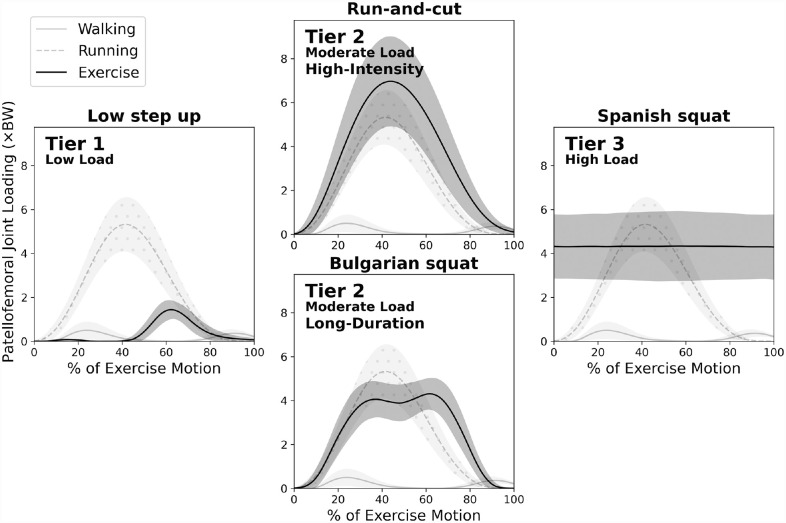
Examples of patellofemoral joint loading over the course of exercise duration. Solid lines indicate group mean; shading indicates ±1 SD. Routine walking and running are graphed in lighter shades for reference. Among the large variety of tier 2 exercises, we present a pair of high-intensity versus long-duration exercises to illustrate their distinctive loading profiles. The long-duration Bulgarian squat (bottom), which also features high knee flexion, exhibits lower loading peak than high-intensity exercises such as run-and-cut (top). Their overall loading levels are comparable because the cumulative loading impulse of the Bulgarian squat (4.4 ×BW·s), which is not visualized in these time-normalized graphs, is higher than that of the run-and-cut (0.8 ×BW·s). BW, body weight.

We found that exercises with a moderate level of patellofemoral joint loading provided the most diverse options for patient-specific rehabilitation planning. Here we propose 2 representative subtypes of exercise to demonstrate such diversity: the “high-intensity” and “long-duration” exercises (Figure 3). Many high-intensity exercises feature both fast speed and high loading peak magnitude, partly due to the moderate correlations between loading peaks and loading rates (Appendix Figure A2). Among the 35 exercises we analyzed, run-and-cut had the second highest loading peak (7.1 ×BW), whereas the 3-second Spanish squat had the highest loading impulse by far among all exercises (13.0 ×BW·s) (Table 1). Remarkably, run-and-cut had a low impulse due to its fast pace and short step time (0.8 ×BW·s), whereas the quasi-static Spanish squat had a moderate loading peak (4.5 ×BW). For this reason, no exercise reached a loading index >0.9, suggesting that unlike Achilles tendon loading,^2^ most exercises do not load the patellofemoral joint with high magnitude and duration simultaneously. Based on our loading index, high-intensity (eg, run-and-cut) and long-duration (eg, Bulgarian squat) rehabilitation exercises may provide an equivalent level of overall loading to the patellofemoral joint and thus can be used as alternative options for prescribing progressive therapeutic load (Figure 3). Yet, we emphasize that these loading ranks and tiers depend on the weights on loading peak, impulse, and rate: namely, the perceived clinical importance among these loading metrics. For example, considering the loading rate–dependent mechanical properties of articular cartilage^24^ and that individuals with patellofemoral pain often struggle to return to running,^9,11,21^ a clinician may perceive loading rate as a primary metric. Accordingly, the clinician may sort these exercises into an alternative order that prioritizes a smooth progression of loading rate, which can be achieved by increasing its weight in the loading index (we give an example in Appendix Figure A3, available online). To assist clinicians with such patient-specific decision making, we present an easy-to-use worksheet in the online Appendix that embeds our group-level loading data, with modifiable loading index metric weights. Researchers can also use this tool to design and compare the efficacy of different data-driven exercise programs and establish clinical standards that best progress patellofemoral loading.

One notable trend among the large collection of moderate- to high-level loading exercises (tier 2 and 3) is that weightbearing exercises involving high knee flexion tended to generate a higher overall level of patellofemoral loading. We defined the force relationship between patellofemoral joint and quadriceps tendon as a function of the knee flexion angle based on a validated mathematical model.^3,4,37^ In this model, the patellofemoral joint force required to balance a certain quadriceps tendon force increases as the knee flexes from 0° to 90°.^37^ Our results support this model definition, because many exercises that involve high knee flexion (Appendix Table A2, available online) are ranked relatively high in overall patellofemoral joint loading according to the loading index (Figure 2). Our findings also match previous biomechanical studies that reported higher patellofemoral joint forces in deeper knee flexion angles.^34^ In contrast, many fast, high-intensity exercises like running and single-leg fast forward hops provide only a low or moderate level of overall loading, despite often being clinically perceived as high-intensity rehabilitation exercises. These findings support the potential benefits of reordering high-intensity and high–knee flexion exercises during rehabilitation in order to smoothly progress loading on the patellofemoral joint under recovery.

Our study quantified patellofemoral loading across a large collection of exercises using a consistent set of mathematical models.^3,4,31,37,42^ As we expected, our loading estimates generally match those reported in previous studies. Our loading peak estimates during walking (0.6 ×BW) compare favorably to the range of values published in the literature (0-0.8 ×BW).^23^ For running, which is an often-studied everyday exercise, Lenhart et al^22^ reported a peak patellofemoral joint force of 5.8 ×BW that compares closely to our estimates of 5.4 ×BW. Patellofemoral joint loading during other dynamic exercises demonstrated more variations between literature reports and our results. Escamilla et al^17^ estimated patellofemoral joint force during single-leg squat up to 4 ×BW, which is notably lower than our estimate (6.9 ×BW). Cleather et al^7^ reported a patellofemoral joint force peak of 4.2 ×BW during the double-leg countermovement jump, which is also lower than our estimate (6.4 ×BW). These disagreements may be partly explained by different movement strategies. For example, Escamilla et al^17^ instructed their participants to squat slowly, whereas our participants squatted at a self-selected pace. Few previous studies have reported knee loading profiles beyond the peak force. With a longer squat duration, the overall patellofemoral loading level reported by Escamilla et al^17^ may have been comparable with ours. Regardless, because we used the same method to estimate patellofemoral loading across all exercises, their loading indices relative to each other should be a robust representation of the loading progression order among these 35 exercises.

These loading index ranks and tiers should be interpreted in light of several limitations. First, our estimates are based on a healthy young adult cohort; thus, these findings do not necessarily represent loading in patients who exhibit patellofemoral pain. We decided to study healthy young adults to eliminate the likelihood of these exercises causing pain in patients, which would make the study impractical and the results nonrepresentative of proper exercise technique. Second, we used a simple marker set for motion capture to simplify our experimental workflow and lessen the burden on our participants. According to our recent study,^32^ the constrained link-segment model we used can mitigate this limitation and reliably estimate lower extremity biomechanics. We believe future research should continue to simplify motion capture (eg, by using markerless techniques that can robustly track motion)^19^ and prioritize expanding biomechanical databases for clinically relevant populations and exercises. A related, third limitation is that by defining a constrained knee with flexion-extension only, our model did not consider the effects of frontal or transverse patellofemoral mobility on loading.^27^ Previous studies reported that patellofemoral forces primarily act in the sagittal plane,^4,26^ which we incorporated in our model. Frontal and transverse kinematics may affect patellofemoral contact area and consequently the mechanical stress,^4,27,29^ which are subject to future research. Fourth, by using the net knee moment to estimate quadriceps and patellofemoral loading required for knee extension, our model neglected the potential effects of coactivation and force production by the knee flexors (eg, hamstrings). A previous study found that quadriceps force may be underestimated up to 1.5 ×BW when neglecting muscle coactivation.^20^ Therefore, our assumption may have lowered the ranking of some squatting or dynamic exercises that require substantial knee flexor activity. Further studies should examine electromyography of the quadriceps and hamstring muscles during these exercises and leverage musculoskeletal simulations to verify whether they would be ranked higher in patellofemoral loading progression, and thus prescribed later during rehabilitation. Fifth, it remains unknown how much loading difference among the exercises is statistically significant. Future research should compare the efficacy of different data-driven exercise programs and determine what levels of patellofemoral loading would be clinically beneficial or detrimental. Sixth, we emphasize again that our choice of loading metric weights was based on our own interpretation of current research concepts.^29,41^ These relative weights can be easily modified in the provided worksheet (Appendix, available online), which will automatically update the loading progression order of the 35 exercises according to the user’s interpretation of overall patellofemoral joint loading. Our loading metric data and mathematical scheme also serve as a benchmark for future research to identify the mechanistic relationships between patellofemoral pain and other pathologies in the anterior knee structures.

## Conclusion

We defined a loading index to quantify, compare, rank, and categorize overall loading levels in the patellofemoral joint across 35 types of commonly prescribed weightbearing rehabilitation exercises and activities of daily living. Overall loading levels varied substantially across the exercises. Most rehabilitation exercises generated a moderate level of patellofemoral joint loading (tier 2). Few weightbearing exercises provided low-level loading (tier 1) that resembles routine walking, whereas only 3 squatting exercises featured high-level loading with both high magnitude and long duration (tier 3).

## Supplemental Material

sj-pdf-1-ajs-10.1177_03635465231175160 – Supplemental material for Patellofemoral Joint Loading Progression Across 35 Weightbearing Rehabilitation Exercises and Activities of Daily Living Supplemental material, sj-pdf-1-ajs-10.1177_03635465231175160 for Patellofemoral Joint Loading Progression Across 35 Weightbearing Rehabilitation Exercises and Activities of Daily Living by Ke Song, Rodrigo Scattone Silva, Todd J. Hullfish, Karin Grävare Silbernagel and Josh R. Baxter in The American Journal of Sports Medicine

sj-xlsx-2-ajs-10.1177_03635465231175160 – Supplemental material for Patellofemoral Joint Loading Progression Across 35 Weightbearing Rehabilitation Exercises and Activities of Daily Living Supplemental material, sj-xlsx-2-ajs-10.1177_03635465231175160 for Patellofemoral Joint Loading Progression Across 35 Weightbearing Rehabilitation Exercises and Activities of Daily Living by Ke Song, Rodrigo Scattone Silva, Todd J. Hullfish, Karin Grävare Silbernagel and Josh R. Baxter in The American Journal of Sports Medicine
