# Supplementary material for: Patellofemoral Joint Loading Progression Across 35 Weightbearing Rehabilitation Exercises and Activities of Daily Living
Source: Am J Sports Med. 2023 Jun 5;51(8):2110–9. doi: 10.1177/03635465231175160 (PMC10315869; doi:10.1177/03635465231175160)
Supplement: sj-pdf-1-ajs-10.1177_03635465231175160 – Supplemental material for Patellofemoral Joint Loading Progression Across 35 Weightbearing Rehabilitation Exercises and Activities of Daily Living [file sj-pdf-1-ajs-10.1177_03635465231175160.pdf]

Patellofemoral Joint Loading Progression Across 35 Weightbearing Rehabilitation Exercises and Activities of Daily Living

Appendix:

Table A1. Exercise data collection order, descriptions and start-stop definitions

Module	Exercise	Description (Leg of interest = Right)	START	STOP
Module #1	Walking	Walk with self-selected speed	Right foot strikes	Right foot lifts off
	Low step up (10-cm)	Step up with right leg, followed by left leg	Right foot lifts off	Left foot touches step
	Low step down (10-cm)	Step down with left leg, followed by right leg	Left foot lifts off	Right foot touches step
	High step up (20-cm)	Step up with right leg, followed by left leg	Right foot lifts off	Left foot touches step
	High step down (20-cm)	Step down with left leg, followed by right leg	Left foot lifts off	Right foot touches step
	Lunge	Lunge forward with right leg, keeping right shank as vertical as possible, then return to neutral position	Right off lifts off	Right foot back neutral
	2-leg countermovement jump	Stand on both legs, perform a countermovement jump, aiming to reach as high as possible	Pelvis starts moving	Pelvis back to neutral
	2-leg drop landing	Drop from 30cm step, land on both legs	Lead foot lifts off	Pelvis stop moving
	2-leg drop vertical jump	Drop from 30cm step, land on both legs, then immediately perform a vertical jump as high as possible	Lead foot lifts off	Both feet lift off again
	2-leg maximal forward jump	Jump forward as far as possible and land on both legs, without losing balance	Either foot strikes	Pelvis stop moving
Module #2	Running	Run with self-selected speed	Right foot strikes	Right foot lifts off
	2-leg squat (60-degree)	Keep feet shoulder-width apart and perform a half squat, touch a chair behind at 60° knee position, then return to neutral	Pelvis starts moving	Pelvis back to neutral
	2-leg squat (full depth)	Keep feet shoulder-width apart, perform a full squat, then return to neutral position		

	1-leg decline squat	Stand on a decline box, keep trunk as upright as possible, and perform a full single-leg squat		
	Sumo squat	Keep feet a bit further apart than shoulder width, with toes pointing out in 45° angle, perform a full squat, then return to neutral		
	3-second Spanish squat	With trunk as upright as possible, squat and hold in a 90° knee position for at least 5 seconds (extract 3 seconds for analysis)	1 second after position set	Hold for 5 seconds
	Run-and-cut	Run towards force plate as fast as possible, reach with right leg, change direction 45° towards left, and run off force plate	Right foot strikes	Right foot lifts off
	1-leg maximal forward hop	Stand on right leg, hop forward as far as possible and land on right leg, without losing balance	Right foot strikes	Pelvis stop moving
	Run-and-stop	Run towards force plate as fast as possible, reach and stop suddenly with right leg, without losing balance	Right foot strikes	
	Sports movement jump	Run forward and jump up with both legs, aiming to reach as high as possible, then land on both legs, one in front of the other	Either foot strikes	
Module #3	1-leg squat (60-degree)	Stand on right leg and perform a half squat, touch a chair placed behind at 60° knee position, then return to neutral	Pelvis starts moving	Pelvis back to neutral
	1-leg squat (full depth)	Stand on right leg and perform a half squat, then return to neutral position		
	Bulgarian squat	With left foot lifted on a 30cm step, perform a full split squat, then return to neutral position		
	1-leg countermovement hop	Stand on right leg, perform a countermovement jump, aiming to reach as high as possible		
	2-leg repetitive forward jumps (regular speed)	Perform small, repeated jumps forward, with knees slightly bent when landing on both legs	Right foot strikes	Right foot lifts off
	2-leg repetitive forward jumps (fast speed)	Repeat the small forward jumps, but at a faster speed		
	2-leg repetitive lateral jumps (regular speed)	Jump sideward, landing with both feet, then jump back to starting position, back and forth between two force plates		
	2-leg repetitive lateral jumps (fast speed)	Repeat the back-and-forth sideward jumps, but at a faster speed		
Module #4	1-leg drop landing	Drop from 30cm step, land on right leg	Lead foot lifts off	Pelvis stop moving
	1-leg drop vertical hop	Drop from 30cm step, land on right leg, then immediately perform a vertical jump as high as possible	Lead foot lifts off	Right foot lift off again
	1-leg repetitive forward hops (regular speed)	Perform small, repeated hops forward with knees slightly bent when landing on right leg only	Right foot strikes	Right foot lifts off

1-leg repetitive forward hops (fast speed)	Repeat the small forward hops, but at a faster speed
1-leg repetitive lateral hops (regular speed)	Hop sideward, landing with right foot only, back and forth between two lines marked 40cm apart on the force plates
1-leg repetitive lateral hops (fast speed)	Repeat the back-and-forth sideward hops, but at a faster speed
Alternating split jumps	Start in a lunge position with right leg in front, perform split squat jumps with the legs alternating back and forth

Supplemental Material

Supplemental Digital Content. Modifiable and sortable worksheet to automatically calculate, rank, and categorize loading index across the 35 rehabilitation exercises and activities. Users can enter weighting factors of their choice for the loading peak, impulse, and rate of patellofemoral joint force. This Supplemental Digital Content is available in the online versions of this article.

[Patellofemoral Loading in 35 Exercises _Supplemental Worksheet.xlsx]

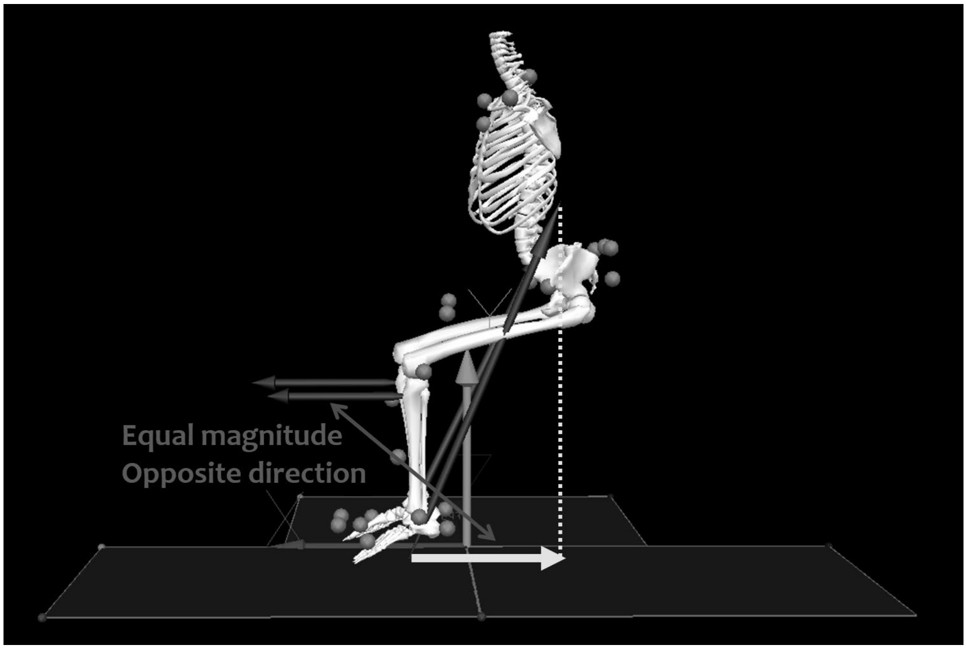

Figure A1. Estimating forces in the resistance belt during the quasi-static Spanish squat. We assumed that force applied to the proximal shank through either strap of the belt (arrows to left) is of equal magnitude and opposite direction to the horizontal ground reaction force under the corresponding foot (arrow to right). We assigned the belt forces at 20% of the shank length from the knee joint towards the ankle joint to match our experimental setup. (Note that the torso segment in our model was for visualization only and had no effect on our lower extremity biomechanical analysis and patellofemoral joint loading computation.)

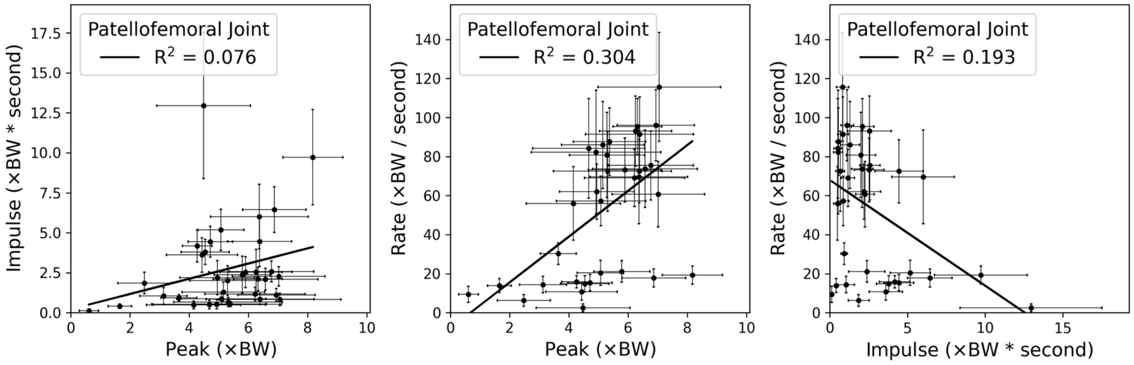

Figure A2. As a secondary analysis, we computed Pearson correlation coefficients squared (R^2) between the patellofemoral joint loading peaks, impulses, and rates across all 35 exercises to explore the interdependencies among these 3 metrics. These scatter plots display each exercise as one scatter point, with a linear regression fit to estimate trends. Loading rates varied in a similar way to loading peaks, demonstrated by the moderate correlations between peaks and rates ($r^2 = 0.304$). However, loading impulses showed few correlations to either loading peaks or loading rates ($r^2 \leq 0.2$).

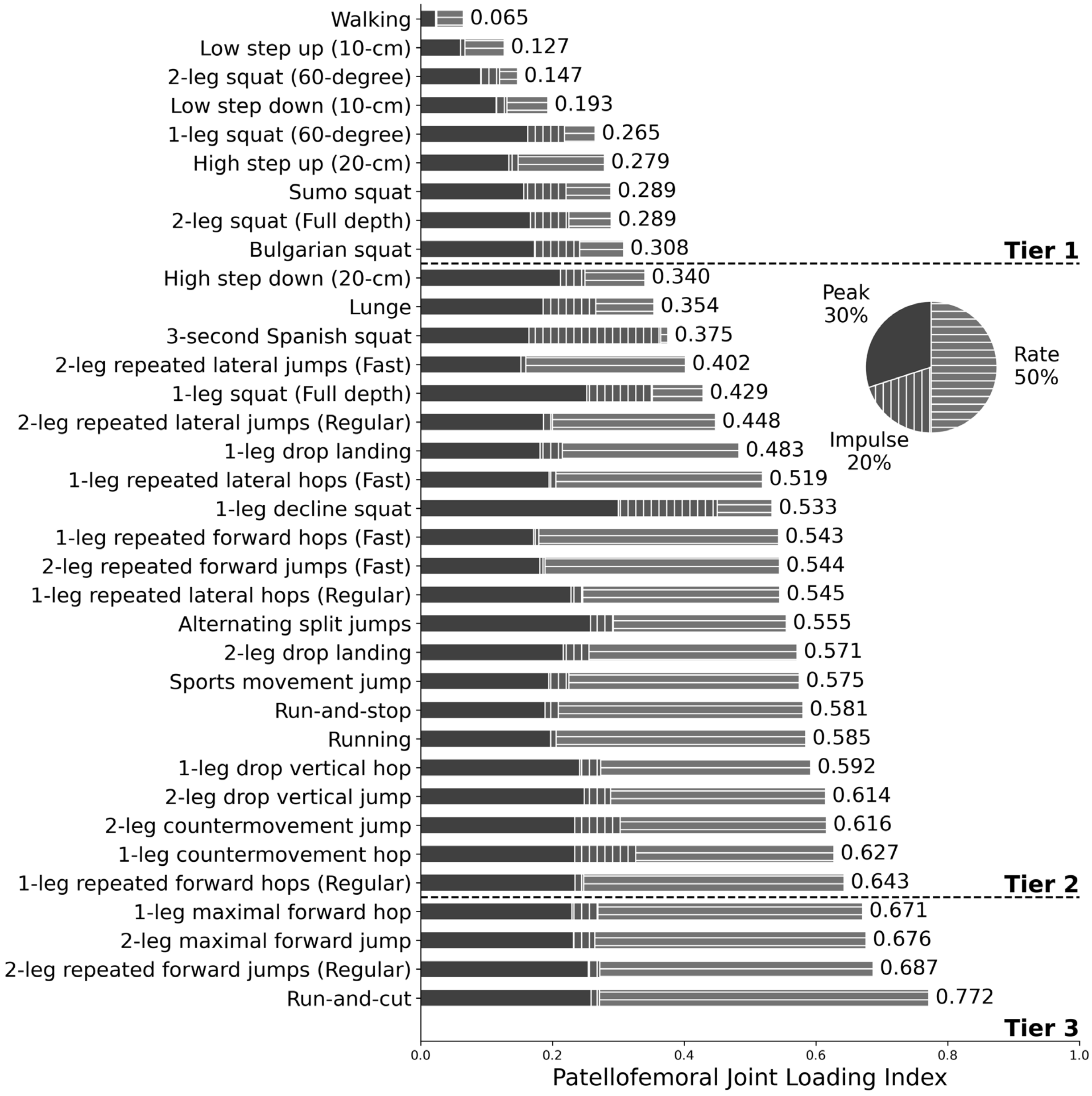

Figure A3. An example of alternative loading index ranks and tiers for the patellofemoral joint across 35 exercises, with a 30% weight for loading peak, 20% for impulse, and 50% for rate. With such alternative weights, Tier 3 and high Tier 2 (loading index > 0.5) along these loading rate-priority ranks are predominantly fast exercises such as running, jumping, and hopping. Their loading rates (horizontal striped) are considerably higher than slower squatting exercises, which are shifted down in Tier 2 or to Tier 1 as a result. The shifted ranks suggest that fast, high-intensity exercises may improve functional recovery for patients with patellofemoral pain who struggle to return to running.

Table A2. Group-level (N = 20) average ± standard deviation (SD) of knee flexion angle metrics across the 35 exercises and activities, listed in ascending order of the patellofemoral loading index.

Loading index tier	Exercise (Unit)	Peak Degree	SD	Impulse Degree*s	SD	Rate Degree/s	SD
**Tier 1**	**Walking**	45.2	5.2	10.4	3.5	329.7	41.7
	**Low step up (10-cm)**	70.5	4.4	48.5	6.0	305.6	28.8
	**2-leg squat (60-degree)**	65.8	9.0	70.6	16.9	117.3	30.4
	**Low step down (10-cm)**	79.7	5.2	54.6	8.7	160.1	26.9
	**High step up (20-cm)**	96.5	4.5	72.1	8.0	398.0	27.8
	**2-leg repetitive lateral jumps (Fast)**	64.0	7.2	13.6	3.1	232.6	87.6
	**1-leg repetitive forward hops (Fast)**	44.9	6.6	8.2	1.8	332.8	50.5
	**2-leg repetitive forward jumps (Fast)**	57.9	10.3	10.1	3.5	455.3	92.3
**Tier 2**	**2-leg repetitive lateral jumps (Regular)**	70.8	9.2	22.8	7.1	325.4	64.8
	**Running**	47.3	4.4	8.3	1.4	451.4	52.4
	**1-leg repetitive lateral hops (Fast)**	48.6	5.2	11.3	3.4	242.0	34.9
	**Run-and-stop**	61.3	9.8	33.0	11.9	504.4	41.8
	**1-leg drop landing**	66.4	8.6	55.3	20.8	444.8	56.8
	**Sports movement jump**	86.0	15.6	53.2	22.3	605.0	71.0
	**1-leg squat (60-degree)**	64.9	7.3	73.5	15.1	104.2	22.9
	**Sumo squat**	118.7	7.7	133.0	25.8	209.8	36.1
	**1-leg repetitive forward hops (Regular)**	51.7	6.8	11.4	3.0	429.5	62.3
	**2-leg squat (Full depth)**	120.9	7.8	119.6	19.1	205.4	37.7
	**1-leg repetitive lateral hops (Regular)**	55.7	6.8	17.2	4.7	289.9	47.7
	**High step down (20-cm)**	97.8	5.0	80.0	19.0	155.6	39.0
	**2-leg drop landing**	92.4	14.6	67.1	24.9	554.6	56.8
	**Bulgarian squat**	102.4	7.6	127.3	23.7	174.5	35.2
	**Run-and-cut**	57.4	8.0	10.3	3.1	433.7	54.2
	**2-leg repetitive forward jumps (Regular)**	73.2	10.7	18.3	7.3	584.2	79.2
	**2-leg maximal forward jump**	99.7	15.4	55.8	16.7	671.0	59.2
	**1-leg maximal forward hop**	70.6	9.0	46.3	19.8	549.6	47.5
	**1-leg drop vertical hop**	68.9	7.0	42.2	12.8	449.9	66.3
	**Lunge**	106.6	8.2	180.6	23.3	309.3	57.9
	**2-leg drop vertical jump**	99.4	12.9	55.2	13.0	556.2	60.3
	**Alternating split jumps**	86.7	10.5	37.9	11.7	354.1	57.2
	**2-leg countermovement jump**	100.5	12.2	108.1	40.7	517.3	77.8
	**1-leg countermovement hop**	75.6	8.5	104.9	27.5	379.6	68.2
**Tier 3**	**1-leg squat (Full depth)**	88.1	8.9	105.7	19.1	145.3	24.6
	**3-second Spanish squat**	83.0	7.5	247.3	22.4	14.2	7.0
	**1-leg decline squat**	84.1	8.4	121.2	36.1	119.2	20.8

Table A3. Group-level (N = 20) average ± standard deviation (SD) of knee extension moment metrics across the 35 exercises and activities, listed in ascending order of the patellofemoral loading index. We normalized the knee moments by subject height (H) times weight (W) (unit: % H×W).

Loading index tier	Exercise (Unit)	Peak %H×W	SD	Impulse %H×W*s	SD	Rate %H×W/s	SD
**Tier 1**	**Walking**	2.4	1.2	0.1	0.6	42.5	12.9
	**Low step up (10-cm)**	4.7	0.9	1.0	0.5	36.4	7.6
	**2-leg squat (60-degree)**	4.6	1.2	4.0	1.3	13.2	4.6
	**Low step down (10-cm)**	7.1	1.3	2.4	1.2	29.4	6.8
	**High step up (20-cm)**	7.5	0.9	1.7	0.5	56.5	7.8
	**2-leg repetitive lateral jumps (Fast)**	8.8	2.5	1.0	0.4	122.5	30.2
	**1-leg repetitive forward hops (Fast)**	12.1	3.6	1.3	0.6	238.3	45.1
	**2-leg repetitive forward jumps (Fast)**	10.2	2.9	1.1	0.4	186.4	53.6
**Tier 2**	**2-leg repetitive lateral jumps (Regular)**	9.9	1.8	1.7	0.6	116.7	18.7
	**Running**	13.7	2.0	1.5	0.3	221.6	26.7
	**1-leg repetitive lateral hops (Fast)**	13.0	3.7	1.6	0.6	184.3	38.7
	**Run-and-stop**	11.1	2.3	2.5	1.4	239.4	60.0
	**1-leg drop landing**	10.2	2.0	4.4	1.8	151.6	26.3
	**Sports movement jump**	9.0	1.7	3.4	1.5	179.1	58.4
	**1-leg squat (60-degree)**	8.4	1.4	7.9	1.8	22.6	6.8
	**Sumo squat**	7.0	1.4	6.9	1.7	22.2	5.2
	**1-leg repetitive forward hops (Regular)**	14.9	2.8	2.1	0.6	224.0	23.2
	**2-leg squat (Full depth)**	7.8	1.5	6.5	1.4	22.1	6.3
	**1-leg repetitive lateral hops (Regular)**	13.8	2.9	2.7	0.7	161.2	27.1
	**High step down (20-cm)**	9.7	1.2	4.5	2.1	33.2	8.8
	**2-leg drop landing**	9.7	1.8	4.2	1.6	132.9	24.6
	**Bulgarian squat**	6.9	1.1	6.5	1.7	27.6	5.8
	**Run-and-cut**	15.0	2.7	1.8	0.6	279.3	47.8
	**2-leg repetitive forward jumps (Regular)**	12.2	2.1	2.1	0.5	182.3	43.8
	**2-leg maximal forward jump**	9.9	1.1	3.4	1.1	180.1	31.7
	**1-leg maximal forward hop**	12.7	2.1	4.9	2.6	252.8	49.3
	**1-leg drop vertical hop**	12.4	2.6	4.1	1.1	160.0	29.2
	**Lunge**	7.6	1.4	8.0	2.5	49.2	14.1
	**2-leg drop vertical jump**	10.4	1.8	4.2	1.0	122.4	20.3
	**Alternating split jumps**	11.0	2.2	3.8	0.8	108.6	25.1
	**2-leg countermovement jump**	10.7	1.6	7.5	2.4	141.3	26.7
	**1-leg countermovement hop**	12.9	2.6	11.9	3.3	167.6	39.8
**Tier 3**	**1-leg squat (Full depth)**	10.3	1.4	11.1	2.2	30.3	6.6
	**3-second Spanish squat**	6.9	2.5	20.0	7.3	3.8	3.0
	**1-leg decline squat**	12.5	1.0	17.5	5.0	32.5	6.1
